# Severe Acute Respiratory Syndrome Coronavirus 2 (SARS-CoV-2) infection among health care workers in Nasarawa State, Nigeria: implications for infection prevention and control measures

**DOI:** 10.11604/pamj.supp.2020.37.1.25767

**Published:** 2020-10-08

**Authors:** Adamu Ishaku Akyala, Jaggu Ruth Awayimbo, Margaret Itake Elayo, Olukemi Titilope Olugbade, Emmanuel Agbadu Akabe, Akinyinka Akinyoade

**Affiliations:** 1 Department of Microbiology, Faculty of Natural and Applied Sciences, Nasarawa State University, Keffi, Nasarawa State, Nigeria,; 2 Nigeria Field Epidemiology and Laboratory Training Programme (N-FELTP), African Field Epidemiology Network, 50 Haile Selassie Street, Asokoro Abuja, Nigeria,; 3 Department of Epidemiology, State Ministry of Health, Lafia, Nasarawa State, Nigeria,; 4 Department of Medical and Health Services, Lagos, Nigeria,; 5 Ministry of Defense, 2, Division Nigeria Army, Ibadan, Nigeria,; 6 Department of Community Health, Obafemi Awolowo University Teaching Hospital (OAUTHC), Ile-Ife, Nigeria

**Keywords:** Clinical characteristics, frontline workers, healthcare workers, transmission

## Abstract

**Introduction:**

health care workers (HCWs) are on the frontline, waging war against SARS-CoV-2 and have a higher risk of infection with exposure to an infected person with SARS-CoV-2. There is a paucity of information on clinical characteristics and infection risk gradient of HCWs with SARS-CoV-2 with the view to marshal preventive measures.

**Methods:**

we conducted a multi-center case series analysis of 648 HCWs who were randomly selected in private and public hospitals across Nasarawa State, managing cases of SARS-CoV-2. Demographic and epidemiological information, were abstracted from electronic medical records of cases from February to July 2020. Throat and Nasopharyngeal swabs and real-time reverse transcriptase-polymerase chain reaction (RT-PCR) tests for SARS-CoV-2 nucleic acid were performed.

**Results:**

overall, 134 of 648 HCWs across health centers in Nasarawa State tested positive for SARS-CoV-2. Eighty male HCWs constituted 30.9% of respondents with a median (interquartile range) age of 36.7 (30.0-47.0) years. Overall, 50 of 134 HCWs (67.5%) with SAR-COV-2 had mild disease. The five most common symptoms amongst cases were fever (67 [90.5%]), myalgia or fatigue (60 [81.1%]), cough (50[67.6%]), sore throat (50 [67.6%]), and muscle ache (50 [67.6%]). Contact with index patients (65 [59.1%]) and colleagues with infection (10 [13.9%]) as well as community-acquired infection (14 [18.9%]) were the main routes of exposure for HCWs.

**Conclusion:**

HCWs in Nasarawa State face an unprecedented occupational risk of morbidity and mortality as a result of SARS-CoV-2. There is need for rapid development of sustainable infection prevention control measures that protect HCWs from the SARS-CoV-2 ongoing pandemic.

## Introduction

An epidemic of respiratory disease caused by severe acute respiratory syndrome coronavirus 2 (SARS-CoV-2) began in Wuhan China and has spread to other countries of the world [1]. In the last 25 years, several highly transmissible respiratory viruses with epidemic potential have emerged. The most significant pandemics of the 20^th^ century, notably, were influenza viruses in 1918, 1957, and 1968. In 2020, a global alert was issued for an emerging, yet unknown illness known as a severe acute respiratory syndrome (SARS) caused by a Novel corona virus subsequently identified as SARS-CoV-2 [2]. Many African countries were not prepared for the Novel SARS-CoV-2 outbreak due to poor and weak healthcare systems, poor disease surveillance and response systems, as well as inadequate and overstretched health facilities and services [3]. The trend of the outbreak established a higher risk of Novel SARS-CoV-2 importation from Europe to Africa than China importation, comparing the rapid spread of the virus in selected sub-Sahara countries than in European countries [4]. It has been established that person-to-person transmission of Novel SARS-CoV-2 in Wuhan city of China occurs and also globally [5]. International emergency concerns were raised by the World Health Organization (WHO), and on January 20^th^, the spread of the novel SARS-CoV-2 virus was declared a pandemic [6,7]. Across affected countries and territories, longer incubation periods and low virulent strain of the virus has increased asymptomatic carriers significantly [8], this is seen in second-generation cases in which patients at their incubation period tend to be asymptomatic and can transmit the virus from one person to another, with healthcare workers especially at risk [9]. In Nigeria, there is a paucity of information regarding transmission and asymptomatic infection rate among at-risk health care workers, and a wider spectrum of hospital surfaces and environmental contamination. SARS-CoV-2 case detection among HCWs, and infection assessment is key in halting and tracking the spread of hospital acquired infections. In our study, our objective was to evaluate the clinical characteristics of SARS-CoV-2 infection and associated risk factors among HCWs in Nasarawa State, North Central, Nigeria.

## Methods

Study design and participants: we revaluated 648 HCWs at selected health care centers designated as isolation and treatment centers for SARS-CoV-2 in Nasarawa State from January 20^th^ to 30^th^ August 2020. We reviewed laboratory and epidemiological demographics using a structured questionnaire from the electronic records at health facilities in the State. First-line health care workers are those that provide direct care to confirmed positive SARS-CoV-2 patients at the isolation and treatment centers. Non-First line health care workers are those that provide care to patients in general at the out-patient departments. We obtained informed consent from all participants and the study was approved by the Nasarawa State Ministry of Health Ethical committee. To assess the prevalence rate of subclinical infection of asymptomatic health care workers we used the reporting guideline of strengthening the Reporting of Observational Studies in Epidemiology (STROBE) [10].

**Clinical laboratory sampling process:** in the diagnosis of SARS-CoV-2, we followed the WHO and the Nigeria Center for Disease Control (NCDC) standard protocol for testing; using nasopharyngeal and throat swab. Samples were taken and immediately placed in a viral transport medium and delivered to the NCDC central Laboratory at Gaduwa, Abuja. Laboratory confirmation of SARS-CoV-2 was carried out by Real-Time Reverse Transcription-Polymerase Chain Reaction. Swabs Sample from environmental surface were obtained within a 100-cm surface from administrative offices, clinics, and other departments.

**Statistical analysis:** epidemiological data obtained at structured interviews were entered in Castor Electronic Data Capture, version 2019. Continuous variables were expressed as medians and ranges and categorical variables were summarized as numbers and percentages. All analyses were done with SPSS version 25.0 (IBM, Armonk, NY, USA). Because of the descriptive nature of our study, sample size calculations and analyses of significance were not done. Results were reported following STROBE guidelines for observational studies. A logistic regression models were used to investigate associations between HCW status and the probability of testing positive for SARS-CoV-2. The results are thus reported as proportions, odds ratios and 95% confidence intervals. P values were 2-sided, with a significance threshold of 0.05

## Results

Data from a total 648 Health Care Workers (HCWs) were abstracted from electronic medical records from the two treatment centers considered in this study. A total 134 HCWs were found to have tested positive for SARS-CoV-2 infection, with a prevalence of 20.7%. Most of the health workers (400, 61.7%) were above 40 years, 359 (30.8%) were physicians and 200 (30.8%) worked in the isolation wards/treatment centers, 100 (15.5%) hypertensive and had 50 mild disease (37.3%). The highest co-morbidities amongst the HCWs were Cardiovascular Disease 200 (30.8) and tuberculosis 128 (19.7) (Table 1). Of the 134 HCWs who tested positive for SARS-CoV-2, 90 (67.2%) were above 40 years, 65 (48.5%) were physicians and 80 (59.7%) worked in the isolation wards/treatment centers. Forty-five (45) HCWs (33.6%) had hypertension as the most common co-morbidity amongst those who tested positive and had mild manifestations (37.3%) of the infection (Table 2). Bivariate analysis showed that HCWs were >40 years, male and had twice the odds of being infected with SARS-CoV-2. Health care workers who were nursing staff had lower odds of getting infected. Factors associated with SARS-CoV-2 infection on multivariate analysis in this study were age > 40 years, job category and working in an Isolation ward/Treatment center (p<0.05) (Table 3).

**Table 1:** socio-demographic and clinical characteristics of infection among health care workers in Nasarawa State (n= 648)

Characteristics	Frequency (%)
**Age (years)**	
<40	44(33.8)
>40	90(67.2)
**Gender**	
Female	54(41.3)
Male	80(59.7)
**Job Category**	
Physician	65(48.5)
Nursing Staff	45(33.6)
Laboratory Personnel	10(7.5)
Administrative Staff	10(7.5)
Others	4(2.9)
**Department**	
Out Patient Department (OPD)	14 (10.4)
Accident & Emergency Unit	40(29.9)
Isolation Wards/Treatment Centers	80(59.7)
**Co-morbidities**	
Tuberculosis	20(14.9)
Cardiovascular Disease	20(14.9)
Hypertension	45(33.6)
Diabetes	30(22.4)
Chronic Obstructive Pulmonary Disease	19(14.2)
**Sign & Symptoms**	
Cough	55 (41.0)
Headache	20(14.9)
Nausea & Vomiting	25(18.7)
Diarrhea	9 (6.7)
Sore Throat	10(7.5)
Fever	15(11.2)
**Disease Severity**	
Mild	50(37.3)
Moderate	40(29.9)
Severe	30 (22.4)
Critical	7 (5.2)
Fatal	7 (5.2)
**Onset of symptom to treatment, median number of days (Inter Quartile Range)**	2.0 (0.0-3.0)
**Incubation period, median (IQR), days**	4.0 (2.0-7.0)

**Table 2:** socio-demographic and clinical characteristics of SARS-CoV-2 in infected HCWs (n= 134)

Characteristics	Frequency (%)
**Age (years)**	
<40	44(33.8)
>40	90(67.2)
**Gender**	
Female	54(41.3)
Male	80(59.7)
**Job Category**	
Physician	65(48.5)
Nursing Staff	45(33.6)
Laboratory Personnel	10(7.5)
Administrative Staff	10(7.5)
Others	4(2.9)
Department	
Out Patient Department (OPD)	14 (10.4)
Accident & Emergency Unit	40(29.9)
Isolation Wards/Treatment Centers	80(59.7)
**Co-morbidities**	
Tuberculosis	20(14.9)
Cardiovascular Disease	20(14.9)
Hypertension	45(33.6)
Diabetes	30(22.4)
Chronic Obstructive Pulmonary Disease	19(14.2)
**Sign & Symptoms**	
Cough	55 (41.0)
Headache	20(14.9)
Nausea & Vomiting	25(18.7)
Diarrhea	9 (6.7)
Sore Throat	10(7.5)
Fever	15(11.2)
**Disease Severity**	
Mild	50(37.3)
Moderate	40(29.9)
Severe	30 (22.4)
Critical	7 (5.2)
Fatal	7 (5.2)
Onset of symptom to treatment, median number of days (Inter Quartile Range)	2.0 (0.0-3.0)
Incubation period, median (IQR), days	4.0 (2.0-7.0)

**Table 3:** factors associated with SARS-CoV-2 infection among health care workers in Nasarawa State

	Frequency (%)	Bivariate			Multivariate		
Characteristics	Health Care Workers with SARS-CoV-2 (n=134)	OR	95%CI	p-value	AOR	95%CI	p-value
**Age (years)**							
<40	44(17.7)	1.0					
≥40	90(22.5)	1.9	1.5-2.4	0.00	1.8	1.4-2.4	<0.05
**Gender**							
Female	54(18.1)	0.6	0.2-1.0	0.115			
Male	80(30.9)	1.5	1.2-2.1	0.006			
**Job Category**							
Physician	65(32.5)	1.0					
Nurses	45(35.2)	0.3	0.2-0.6	0.008			
Lab Personnel	10(8.3)	0.8	0.5-1.0	0.046			
Admin Staff	10(10.0)	0.7	0.4-1.3	0.084			
Others	4(4.0)	0.9	0.7-1.4	0.042	0.7	0.4-0.8	0.000
**Department**							
Out Patient Department (OPD)	14 (6.1)	1.0	0.8-1.4	0.5			
Accident & Emergency	40(18.3)	0.9	0.5-1.1	0.11			
Isolation Wards /Treatment Center	80(40.0)	0.8	0.4-2.1	0.845	1.4	1.1-1.8	0.02
**Disease Severity**							
Mild	50(39.1)	1.0					
Moderate	40(20.0)	0.4	0.3-0.7	0.009			
Severe	30 (30)	0.7	0.6-1.1	0.035			
Critical	7 (5.3)	0.9	0.6-1.4	0.073			
Fatal	7 (7)	1.0	0.8-1.5	0.031	0.7	0.5-0.9	0.006
**Rapid Response Responsibility**							
First-line	90(22.5)	1.0	0.6-1.1	0.124	1.7	1.3-2.3	<0.05
None First Line	44(17.7)	0.8					

## Discussion

This study looked at the distribution of socio-demographic characteristics and clinical signs of SARS-CoV-2 amongst HCWs in Nasarawa State Nigeria. Of the healthcare workers considered in this study, a fifth tested positive for SARS-CoV-2. Most of the HCWs in this study were physicians, most studies that have considered SARS-CoV-2 infections in HCWs had a higher proportion of nurses compared to other HCWs [11,12]. Most of the affected HCWs who got infected, worked in Out Patient Units as Primary Care Givers, this is similar to findings which showed that Primary Care HCWs are more at risk of infectious diseases [13,14]. Up to a third of the HCWs, and a sixth of those with SARS-CoV-2 had underlying disease, this finding is similar to a study that showed that up to the same proportion of cases with the infection had underlying disease [14]. Similarly, as with the trend in affected countries, more of the underlying conditions among HCWs in this study were cardiovascular in origin [14]. The range of the interval between symptom onset to treatment, and median incubation period of the infection in this study was similar to a study conducted in China [15]. The trends of manifestation of clinical signs and symptoms of the SARS-CoV-2 infection among HCWs in this study were also similar to the infection patterns found in other studies, with cough being the most common symptom, followed by gastrointestinal symptoms [15,16]. Only about 1 in 12 cases had fever which was a little different from previous studies conducted that showed that more than a third of cases had fever [11,15]. Studies have shown that implementation of prevention and control (IPC) during outbreaks of infectious disease such as Ebola Virus Disease, Severe Acute Respiratory Syndrome (SARS), Middle Eastern Respiratory Syndrome (MERS) and now SARS-CoV-2 or other infectious diseases are of great importance in ensuring the safety, wellbeing and protection of healthcare [16]. The prevalence of SARS-CoV-2 is very high in this study compared to other studies in areas with early onset SARS-CoV-2 infections. A study in China documented 3.8% of HCWS were said to have contracted the infection, this was lower than findings from this study. In the same study in China, 1 in 9 cases in HCWs and were classified as severe [17]; in our study, up to 1 in 5 HCWs got infected with SARS-CoV-2. Factors associated with SARS-CoV-2 infection in this study included being more than 40 years of age and being male. This is similar to findings from current global research conducted and most recent global findings which showed that most persons who got infected were males compared to females, and were of older age [17-19].

## Conclusion

This study established that, at the early stage of the COVID-19 pandemic, HCWs in Nasarawa State of Nigeria were at high risk of infection SARS-CoV-2. In the context of SARS-CoV-2, HCWs face an unprecedented occupational risk of morbidity and mortality. Lack of and/or inadequate PPE, exposure to infected patients, work overload, poor infection control, and preexisting medical conditions put HCWs at risk for nosocomial SARS-CoV-2 infection. Further studies are needed to inform the development of efficacious infection control measures. There is a need for rapid development of sustainable measures that protect HCWs from the pandemic.

### What is known about this topic

- HCWs face an unprecedented occupational risk of morbidity and mortality from infectious diseases;
- Lack of and/or inadequate PPE, exposure to infected patients, work overload, poor infection; control, and preexisting medical conditions put HCWs at risk for nosocomial SARS-CoV-2 infection.

### What this study adds

- The study sheds lights on the exposure and burden of COVID-19 amongst HCWs across health centers in Nasarawa State;
- The study provides details of factors associated with COVID-19 among HCWs in health centers in Nasarawa State such as: associated risk factors, job profile and workplace location.
